# Increased heme-oxygenase 1 expression in mesenchymal stem cell-derived adipocytes decreases differentiation and lipid accumulation via upregulation of the canonical Wnt signaling cascade

**DOI:** 10.1186/scrt176

**Published:** 2013-03-12

**Authors:** Luca Vanella, Komal Sodhi, Dong Hyun Kim, Nitin Puri, Mani Maheshwari, Terry D Hinds, Lars Bellner, Dov Goldstein, Stephen J Peterson, Joseph I Shapiro, Nader G Abraham

**Affiliations:** 1 Joan C. Edwards School of Medicine, Marshall University, 1600 Medical Center Drive, Huntington, WV, 25701-3655, USA; 2 Department of Drug Science, Section of Biochemistry, University of Catania, Viale Andrea Doria, 6, 95125 Catania, Italy; 3 Department of Physiology and Pharmacology, University of Toledo, 3000 Arlington Avenue, Toledo, OH, 43614-2598, USA; 4 Department of Medicine, New York Medical College, Munger Pavilion, Valhalla, NY, 10595, USA; 5 Brooklyn Fertility Center, Columbia University, 55 Central Park West, New York, NY 10023, USA

## Abstract

**Introduction:**

Heme oxygenase (HO), a major cytoprotective enzyme, attenuates oxidative stress and obesity. The canonical Wnt signaling cascade plays a pivotal role in the regulation of adipogenesis. The present study examined the interplay between HO-1and the Wnt canonical pathway in the modulation of adipogenesis in mesenchymal stem cell (MSC)-derived adipocytes.

**Methods:**

To verify the role of HO-1 in generating small healthy adipocytes, cobalt protoporphyrin (CoPP), inducer of HO-1, was used during adipocyte differentiation. Lipid accumulation was measured by Oil red O staining and lipid droplet size was measured by BODIPY staining.

**Results:**

During adipogenesis *in vitro*, differentiating pre-adipocytes display transient increases in the expression of genes involved in canonical Wnt signaling cascade. Increased levels of HO-1 expression and HO activity resulted in elevated levels of β-catenin, pGSK3β, Wnt10b, Pref-1, and shh along with increased levels of adiponectin (*P *< 0.05). In addition, induction of HO-1 resulted in a reduction in C/EBPα, PPARγ, Peg-1/Mest, aP2, CD36 expression and lipid accumulation (*P *< 0.05). Suppression of HO-1 gene by siRNA decreased Wnt10b, pGSK3β and β-catenin expression, and increased lipid accumulation. The canonical Wnt responsive genes, IL-8 and SFRP1, were upregulated by CoPP and their expression was decreased by the concurrent administration of tin mesoporphyrin (SnMP), an inhibitor of HO activity. Furthermore, knockdown of Wnt10b gene expression by using siRNA showed increased lipid accumulation, and this effect was not decreased by concurrent treatment with CoPP. Also our results show that blocking the Wnt 10b antagonist, Dickkopf 1 (Dkk-1), by siRNA decreased lipid accumulation and this effect was further enhanced by concurrent administration of CoPP.

**Conclusions:**

This is the first study to demonstrate that HO-1 acts upstream of canonical Wnt signaling cascade and decreases lipogenesis and adipocyte differentiation suggesting that the HO-1 mediated increase in Wnt10b can modulate the adipocyte phenotype by regulating the transcriptional factors that play a role in adipogenesis. This is evidenced by a decrease in lipid accumulation and inflammatory cytokine levels, increased adiponectin levels and elevation of the expression of genes of the canonical Wnt signaling cascade.

## Introduction

Human bone marrow-derived mesenchymal stem cells (MSCs) are multipotent cells that have the potential to differentiate into a variety of cell types including adipocytes [1-5]. MSC-derived adipocyte differentiation and dysregulation of adipogenesis is implicated in the pathogenesis of diseases such as metabolic syndrome [4]. Enhanced adipogenesis with adipocyte hypertrophy is one of the leading causes of adipose tissue hypoxia, inflammation, and dysfunction [6]. Hence, the elucidation of the mechanisms that regulate commitment of MSCs towards adipogenic fate may offer a portal to the development of treatment for metabolic syndrome and its related vascular complications.

Adipogenesis begins with the commitment of MSCs to the adipocyte lineage, followed by terminal differentiation of pre-adipocytes to mature adipocytes [5,7]. Fat tissue-derived adipocytes express several regulatory proteins such as Wnts and β-catenin, as well as Sonic hedgehog (Shh), which potentially works upstream of these known differentiation factors to induce osteogenesis in MSCs [8]. Wnts regulate gene expression through either the canonical (β-catenin-dependent) or the non-canonical (β-catenin-independent) pathway [9,10]. The canonical Wnt signaling pathway controls cell proliferation, cell survival and cell fate. Wnt ligands are secreted glycoproteins that function in a paracrine and autocrine manner. Among the Wnt ligands identified, Wnt10b has been shown to be a crucial factor in the activation of the canonical pathway and inhibition of adipogenesis [11,12]. Adipose tissue-specific transgenic over-expression of Wnt10b leads to a significant decrease in adiposity and resistance to a high-fat diet in mice [13]. The canonical Wnt pathway relies on stabilization of β catenin. The Wnt/β catenin signaling pathway affects cellular functions by regulating both β catenin levels and subcellular localization [14].

An increase in Wnt/β-catenin signaling inhibits the adipogenic transcription factor CCAAT/enhancer binding protein (C/EBPα) and the peroxisome proliferator activator receptor (PPARγ) [11,15-17]. Adipocyte differentiation is an ordered multistep process requiring the sequential activation of several groups of transcription factors, including CCAAT/enhancer-binding protein (C/EBPα) gene family and peroxisome proliferator activated receptor-γ (PPAR-γ) [1,18]. C/EBPα and PPARγ are involved in the growth arrest that is required for adipocyte differentiation. Pre-adipocyte factor-1 (Pref-1) belongs to the Notch family of epidermal growth factor-like repeat-containing proteins and has been shown to participate in maintaining pre-adipose phenotype [19]. Pref-1 is an inhibitor of adipocyte differentiation, hence a decrease in Pref-1 expression is observed during differentiation of adipocytes [20]. The paternally expressed 1 (Peg-1)/Mesoderm-specific transcript (Mest) [21], when upregulated, results in the enlargement of adipocytes during adipose tissue expansion [22]. On accumulation of triglycerides, the levels of Peg-1/Mest [22] are increased with a concomitant signal to pre-adipocytes to enlarge in order to accommodate more triglycerides. Adipocyte enlargement is associated with an increase in the levels of TNFα, IL-1, IL-6 and increased insulin resistance [23-26]. Hedgehog signaling exerts its pleiotropic effects through regulation of the cell cycle, direction of cell differentiation, and alteration of cell survival [27]. Conversely, Shh signaling represses adipogenic differentiation in pre-adipocytes [28].

Heme oxygenase-1 (HO-1) is a stress response gene critical for bone marrow cell differentiation [29-31]. A porphyrin structure within cyanocobalamine enables it to induce *HO-1 *gene expression by facilitating its binding to the porphyrin binding domain of the *HO-1 *gene. Induction of HO-1 enhances cell survival and moderates diabetes and obesity [32]. Induction of HO-1 gene expression *in vivo *and in cell culture results in an increase in pre-adipocytes, a reduction in the number of enlarged adipocytes, and an increase in small adipocyte and adiponectin levels [33]. A decrease in HO-1 expression results in increased insulin resistance and adiposity in Zucker rats and obese mice [34]. Additionally, induction of HO-1 in adipocyte cell culture is associated with increased adiponectin levels and decreased pro-inflammatory cytokines, TNFα and IL-6 [35,36].

The goal of this study was to elucidate the role of *HO-1 *gene expression on adipogenesis and clarify the role of Wnt10b and its dependent genes in this process. Induction of *HO-1 *gene expression and HO activity decreased lipid deposition and inflammatory cytokine levels, increased adiponectin levels and elevated the expression of genes of the canonical Wnt signaling cascade. These novel findings demonstrate that increased levels of HO-1 appear crucial in modulating the phenotype of adipocytes to express canonical downstream signaling proteins.

## Materials and methods

### Differentiation of human bone marrow-derived MSCs into adipocytes

Frozen bone marrow mononuclear cells were purchased from Allcells (Allcells, Emeryville, CA, USA). After thawing, mononuclear cells were resuspended in an α-minimal essential medium (α-MEM, Invitrogen, Carlsbad, CA, USA) supplemented with 10% heat-inactivated FBS (Invitrogen) and 1% antibiotic/antimycotic solution (Invitrogen). The cells were plated at a density of 1 to 5 × 10^6 ^cells per 100-cm^2 ^dish. The cultures were maintained at 37°C in a 5% CO_2 _incubator and the medium was changed after 48 h and every 3 to 4 days thereafter. When the MSCs were confluent, the cells were recovered by the addition of 0.25% trypsin/ethylenediaminetetraacetic acid (EDTA) (Invitrogen). MSCs (passage 2 to 3) were plated in a 75-cm^2 ^flask at a density of 1 to 2 × 10^4 ^cells and cultured in α-MEM with 10% FBS for 7 days. The medium was replaced with adipogenic medium, and the cells were cultured for an additional 14 days as described previously [37]. Human MSCs, passage 3, were cultured in the presence of the HO-1 inducer cobalt protoporphyrin (CoPP) (5 μM) and with the HO activity inhibitor tin (Sn^4+^)-mesoporphyrin (SnMP) (5 μM), which were administered every 2 days.

### HO activity measurement

Heme oxygenase activity was measured in hMSCs by carbon monoxide (CO) production in cellular homogenates. Briefly, hMSCs were homogenized in Sucrose (255 mM)-Tris hydrochloride (20 mM) buffer (pH 7.4) with NP-40 (1% w/v), EDTA (1 mM), phenylmethylsulfonyl fluoride (PMSF) (1 mM) and mammalian protease inhibitor cocktail (5% v/v). After homogenization, samples were centrifuged, at 6000 × g for 30 minutes at 4°C, and the supernatant collected for measurement of HO activity; 100 μg protein/sample was incubated, in gas-sealed vials, in Sucrose-Tris buffer along with nicotinamide adenine dinucleotide phosphate-oxidase (NADPH) (1 mM) and excess heme (40 μM), in both the absence and the presence of SnMP (2 μM). Samples were incubated in a water bath, in the absence of light, at 37°C for 60 minutes, after which, the HO reaction was stopped by placing the samples in ice. CO generation was quantitated in the headspace using gas chromatography/mass spectrometry (GC/MS), as previously described [38], using C^13^O^16 ^as an internal standard. Results are expressed as HO-dependent CO generation by subtracting the amount of CO in the presence of SnMP. CO generated is expressed as pmoles/mg protein/hour.

### Effect of CoPP on adipogenesis

To measure the effect of increased HO-1 expression on MSC-derived adipocyte differentiation, cells were treated with 0.5, 1.0, 2.0, 5.0, and 10.0 μM of CoPP every 4 days. After 14 days, cells were stained with Oil Red O solution.

### Oil Red O staining

Staining was performed using 0.21% Oil Red O in 100% isopropanol (Sigma-Aldrich, St. Louis, MO, USA). Briefly, adipocytes were fixed in 10% formaldehyde, stained with Oil Red O for 10 minutes, rinsed with 60% isopropanol (Sigma-Aldrich), and the Oil Red O eluted by adding 100% isopropanol for 10 minutes and the optical density (OD) measured at 490 nm, for 0.5 sec reading.

### Measurement of lipid droplet size

After induction of adipogenesis, lipid droplets were stained with 2 μM boron-dipyrromethene (BODIPY) 493/503 (Molecular Probes, Eugene, OR, USA) [39]. Cell size was measured using an ImagePro Analyzer (MediaCybernetics, Inc., Bethesda, MD, USA). The classification of the size of lipid droplets was based on size by area (pixels).

### Cell viability test by lactic dehydrogenate assay (LDH)

We followed the manufacturer's protocol (LDH Assay kit, Cayman, Ann Arbor, MI, USA). Briefly, hMSC and adipocytes at day 14 were plated in 96-well plates for 1 day. Next day, cell layers were washed twice with PBS, and then cells were treated with various concentrations of CoPP (0 to 10 μM). After incubation for 24 h, and addition of 100 μl of reaction mixture to each well, cells were incubated for 4 hours at 37°C and 5% CO_2 _in a humidified incubator. Absorbance was measured in the 96-well microplate using a microplate reader at 490 nm with 650 nm as the reference wavelength, and the percentage of LDH release for each sample was normalized according to the absorbance reading from samples treated with 0.5% Triton X-100. All analyses were replicated eight times.

### Cytokine array and adiponectin

TNFα and adiponectin (high molecular weight, HMW), were measured as previously described [25,40] by Cytokine SearchLight Infrared arrays (Pierce Biotechnology, Inc., Woburn, MA, USA).

### HO-1 siRNA transfection

Cells were treated with three different predesigned siRNAs of the HO-1 gene (SASI_ Hs01_00035068, SASI_Hs01_00035065 and SASI_Hs01_00035067 from Sigma-Aldrich, St. Louis, MO, USA). According to the manufacturer's protocol, adipogenic media containing siRNA using NTER (Sigma-Aldrich) was replaced every 48 h. Briefly, a nanoparticle solution was incubated with 10 nM siRNA. After 20 minutes cells were treated with siRNA solution during adipogenesis, which was halted after 10 days.

### Measurement of MSC-derived adipocyte signaling molecules

Cells were maintained at -80°C until required for assay. Frozen cells were pulverized and placed in a homogenization buffer (10 mM phosphate buffer, 250 mM sucrose, 1 mM EDTA, 0.1 mM PMSF and 0.1% tergitol, pH 7.5). Homogenates were centrifuged at 27,000 × g for 10 minutes at 4°C. The supernatant was isolated and protein levels were assayed (Bradford Method). The supernatant was used for measurement of HO-1, Wnt10b, β-catenin, Pref-1, C/EBPα, Peg-1/Mest, pGSK3β, shh, PPARγ and β-actin levels as described previously [25,41]. β-Actin was used to ensure adequate sample loading for all western blots.

### Quantitative real-time PCR analysis

Total RNA was extracted from differentiated human mesenchymal stem cells using 5-Prime PerfectPure RNA Tissue Kit (Fisher Scientific Company, LLC, Wilmington, DE, USA). Total RNA was read on a NanoDrop 2000 spectrophotometer (Thermo Fisher Scientific) and cDNA was synthesized using High Capacity cDNA Reverse-Transcription Kit (Life Technologies, Grand Island, NY, USA). PCR amplification of the cDNA was performed by quantitative real-time PCR using TrueAmp SYBR Green qPCR SuperMix (Smart Bioscience, Philadelphia, USA). The thermocycling of IL-8 and Secreted frizzled-related protein 1 (SFRP1) protocol consisted of 5 minutes at 95°C, 40 cycles of 15 sec at 95°C, and 30 sec at 60°C, and finished with a melting curve ranging from 60 to 95°C to allow distinction of specific products. Normalization was performed in separate reactions with primers to 18S mRNA (TTCGAACGTCTGCCCTATCAA and ATGGTAGGCACGGCGACTA).

### Statistical analyses

Statistical significance (*P *< 0.05) of differences between experimental groups was determined by the Fisher method for analysis of multiple comparisons. For comparison between treatment groups, the null hypothesis was tested by either single-factor analysis of variance (ANOVA) for multiple groups, or the unpaired *t*-test for two groups, and the data are presented as mean ± standard error (SE).

## Results

### The effect of adipogenesis on HO-1 expression

**Figure 1:**
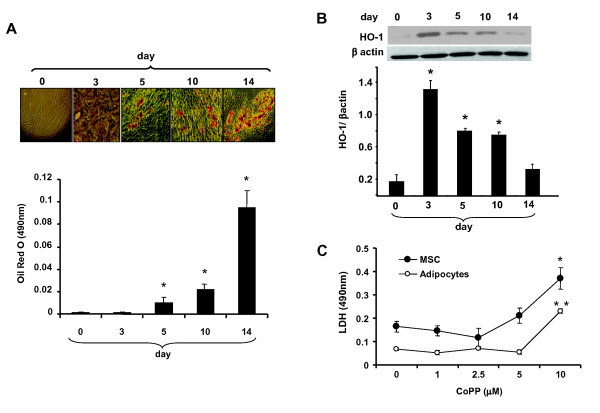
**Expression of heme oxygenase-1 (HO-1), with time, on adipogenesis**. (**A**) Pictures of lipid droplets of a representative experiment at days 3, 5, 10, and 14. Adipogenesis was measured as the relative absorbance of Oil Red O as described in Materials and methods (mean ± SD, *P < 0.05 vs control). (**B**) Western blot of HO-1 and actin in mesenchymal stem cell (MSC)-derived adipocytes from day 0 to day 14. Representative immunoblots and densitometry analysis are shown. Data are expressed as means ± SD; *n *= 4; *P < 0.05. (**C**) Lactic dehydrogenate (LDH) assay done to study the cytotoxicity at increasing concentrations of cobalt-protoporphyrin IX (CoPP). Data are expressed as means ± SD; *n *= 8, *P < 0.05 vs MSC control, **P < 0.05 vs adipocytes control.

The temporal sequence of HO-1 expression was determined over a 14-day period in MSC-derived adipocytes. The basal levels of HO-1 expression increased during MSC adipogenic culture growth and peaked at day 3 (Figure 1B). This was followed by gradual decrease in HO-1 expression, while lipid droplet area gradually increased over a period of 14 days (Figure 1A).

### The effect of CoPP on cell viability during adipogenesis by LDH assay

Detection of cell membrane integrity is a rapid and simple approach to determine cell viability by measuring cellular LDH leakage in damaging cells. Our results showed that CoPP treatment in MSCs and adipocytes at day 14 have no cytotoxic effects at concentrations up to 5 μM (Figure 1C). However, MSCs and adipocytes showed significant toxicity at a 10 μM concentration of CoPP (*P *< 0.05).

### The effect of CoPP on HO-1 expression, HO activity and cytokine levels during adipogenesis

**Figure 2:**
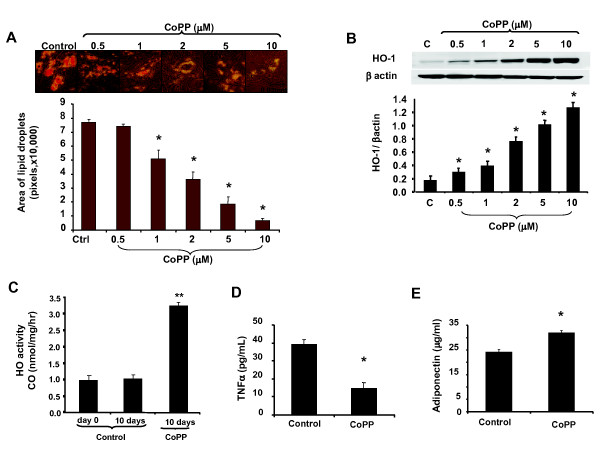
**Effect of cobalt-protoporphyrin IX (CoPP) on lipid accumulation, heme oxygenase-1 (HO-1) expression and activity, and cytokine levels**. (**A**) Pictures of lipid droplets of a representative sample in the presence of increasing CoPP concentrations. Adipogenesis was measured as the relative absorbance of Oil Red O at day 14 after inducing adipogenesis as described in Materials and methods (mean ± SD, *P < 0.05 vs control). (**B**) Western blot analysis of HO-1protein expression in response to increasing concentration of CoPP treatment (0.5, 1.0, 2.0, 5.0, 10.0 μm). Data are expressed as means ± SD; *n *= 4 (*P < 0.05 vs control). (**C**) HO activity measured by CO formation (**P < 0.05 vs control). (**D-E**) Effect of CoPP on cytokine levels in control and cells treated with CoPP. CoPP was added every 2 days for 2 weeks, and cultured media samples were obtained immediately before the media was changed. Results are calculated as pg/ml of cultured media for TNF-α (**D**) and μg/ml for adiponectin (**E**) (*P < 0.05 versus control).

We examined the effect of CoPP on lipid accumulation after 14 days, using standard culture conditions by measuring Oil Red O-stained lipid droplet area (Figure 2A). The level of cells with Oil Red O-stained lipid droplets decreased as CoPP concentration increased. CoPP decreased lipid accumulation in a concentration-dependent manner. Oil Red O staining was barely detectable at the highest concentration of CoPP (10 μM), although untreated MSC-derived adipocyte progenitor cells were loaded with lipid droplets (control). Quantification of Oil Red O-stained cells showed an increase in the number of adipocytes in the absence of CoPP (77,093 ± 943 pixels) compared with the presence of CoPP (54,376 ± 5,366 and 3,526 ± 368 pixels) at 1 and 10 μM, respectively. The expression of HO-1 in the presence of CoPP increased in a dose-dependent manner (Figure 2B), which was consistent with our results showing decreased lipogenesis with increased concentrations of CoPP (Figure 2A). HO activity, as measured by CO release, was increased in the presence of CoPP compared to the control (Figure 2C; *P *< 0.05). HO activity increased reaching a maximum (*P *< 0.05) at day 10 and remained elevated. This was in contrast to the control where HO activity remained at the same level as day 0. One possible explanation of this discrepancy between HO-1 expression and HO activity could be differential effects of progressing adipogenesis on HO-1 expression and activity. Other investigators [42,43] have reported redox imbalances during the process of adipogenesis. We have previously shown that oxidative stress can induce HO expression while suppressing HO activity [44] (Figure 2C).

Adipose cell enlargement is associated with increased secretion of cytokines, which impairs the differentiation of pre-adipocytes and reduces adiponectin secretion. We examined the levels of TNFα in the conditioned media of CoPP-treated MSC-derived adipocytes, and found that TNFα levels were significantly decreased (Figure 2D; *P *< 0.001) at day 14. In contrast, CoPP increased adiponectin levels were increased subsequent to CoPP treatment when compared to controls at day 14 (*P *< 0.05; Figure 2E).

### Effect of CoPP and SnMP on adipogenesis and distribution of lipid droplet size, stained by BODIPY

**Figure 3:**
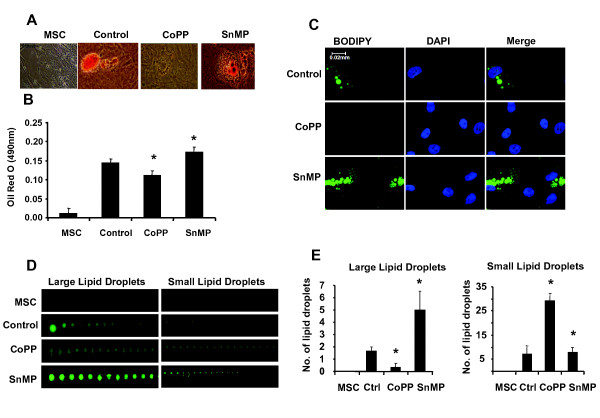
**Effect of CoPP and SnMP on MSC-derived adipocyte cell differentiation**. (**A-B**) Effect of CoPP and SnMP on adipogenesis. Adipogenesis was measured as the relative absorbance of Oil Red O at day 14 after inducing adipogenesis as described in Materials and Methods. (**C-E**) Effect of CoPP and SnMP on lipid droplet size. Lipid droplets were stained with boron-dipyrromethene (BODIPY) and the sizes of droplets were measured using Image Pro Analyzer (ver. 6.2, Media Cybernetics, Inc., MD, USA), *P < 0.05 vs control).

After 14 days, the number of lipid droplets stained with Oil Red O was lower after CoPP treatment when compared to control (*P *< 0.05; Figure 3A-B). In contrast, HO activity inhibitor, SnMP treatment, resulted in higher levels of Oil Red O staining when compared to control (Figure 3A-B). To confirm that increased levels of HO-1 resulted in decreased lipid accumulation, we stained lipid droplets with BODIPY/4',6-diamidino-2-phenylindole (DAPI) (Figure 3C). CoPP treatment decreased the number of very large lipid droplets (*P *< 0.05) and increased the number of small lipid droplets when compared with control (Figure 3D). In contrast, SnMP significantly increased the number of large lipid droplets (*P *< 0.05) and decreased the number of small lipid droplets, compared with either culture treated with CoPP or control, indicating that this effect is mediated by increased HO activity (Figure 3D). The results were quantified and are shown in Figure 3E.

### Effect of CoPP and SnMP on CD36 expression

**Figure 4:**
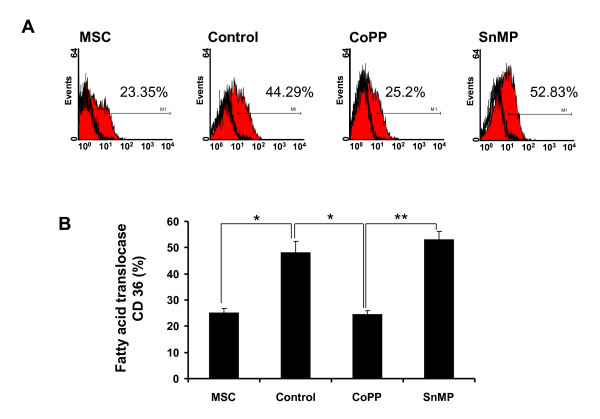
**Effect of cobalt-protoporphyrin IX (CoPP) and tin (stannic)-mesophorphyrin IX (SnMP) on the surface marker CD36**. Membrane antigen expression of CD36 on h-mesenchymal stem cells (MSCs) and MSC-derived adipocytes was analyzed by a fluorescence-activated cell sorter (FACS). Cells were treated with adipogenic media and collected after 14 days. Data are expressed as means ± SD (*P < 0.05, **P < 0.05).

CD36, a fatty acid translocase, which mediates the transfer of fatty acids (FAs) into the cell [45], was measured in MSCs and MSC-derived adipocytes treated with either CoPP (5 μM) or SnMP (5 μM). Cells were treated with adipogenic media and collected after 14 days. As seen in Figure 4A, during lipogenesis CoPP decreased CD36 expression (25.21 ± 1.67%) compared to control (44.29 ± 4.33%), while SnMP increased CD36 expression (52.83 ± 3.12%). The results are quantified in Figure 4B and show that CoPP decreased CD36 levels (*P *< 0.05) compared to control indicating decreased fatty acid uptake in adipocytes by upregulation of HO-1. As expected, SnMP restored CD36 levels to those seen in control, suggesting that increased HO-1 expression and activity has anti-adipogenic response on adipocytes.

### Effect of siRNA HO-1 on adipogenesis

**Figure 5:**
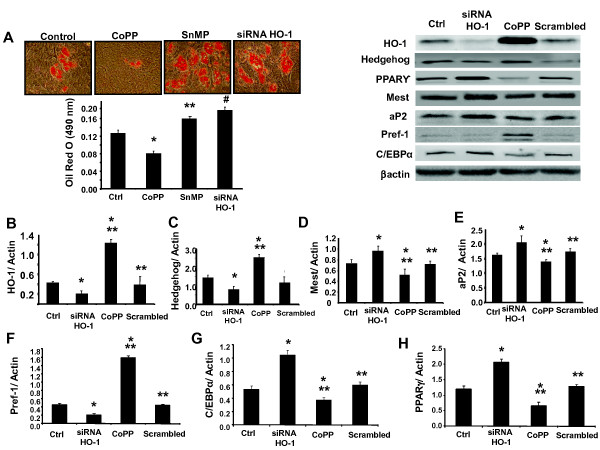
**Effect of heme oxygenase-1(HO-1) induction and suppression on adipogenic markers**. (**A**) Effect of cobalt-protoporphyrin IX (CoPP), tin (stannic)-mesophorphyrin IX (SnMP) and siRNA HO-1 on lipid accumulation. Lipogenesis was measured as the relative absorbance of Oil Red O at day 14 after inducing adipogenesis as described in Materials and methods (mean ± SD, *P < 0.05 vs control, **P < 0.05 vs control, ^#^*P *< 0.05 vs control). (**B-H**) Effect of CoPP, SnMP and siRNA HO-1 on HO-1 expression, and adipogenic marker expression in mesenchymal stem cell (MSC)-derived adipocytes. (**B**) Densitometry analysis of HO-1expression (*P < 0.01 vs control, **P < 0.05 vs siRNA HO-1). (**C-H**) Densitometry analysis of Sonic hedgehog (shh), mesoderm-specific transcript (Mest), fatty acid binding protein (aP2), Pre-adipocyte factor-1 (Pref-1), adipogenic transcription factors CCAAT/enhancer binding protein a (C/EBPα), peroxisome proliferator-activated receptor (PPAR)γ levels, respectively (*P < 0.01 vs control, **P < 0.05 vs siRNA HO-1).

Upregulation of HO-1 by CoPP decreased lipid accumulation, which was measured as the relative absorbance of Oil Red O. To selectively assess the role of HO-1 on adiposity, HO-1 siRNA was added to the cell culture. Lipid accumulation was increased after HO-1 siRNA treatment as compared to MSCs treated with CoPP (Figure 5A; *P *< 0.05). Densitometry analysis showed that CoPP treatment increased HO-1 expression in MSC-derived adipocytes compared to the control, and this effect was reversed by siRNA HO-1 (Figure 5B; *P *< 0.01). The protein expression of Shh increased with CoPP treatment and was reversed by siRNA HO-1 (Figure 5C). Pref-1, an excellent marker for pre-adipocytes, which is extinguished during adipocyte differentiation [19], was increased by CoPP treatment and was decreased by siRNA HO-1 (Figure 5G). Upregulation of HO-1 by CoPP treatment decreased levels of the adipogenic markers Peg-1/Mest, aP2, C/EBPα and PPARγ in MSC-derived adipocytes, and this effect was reversed by transduction with HO-1 siRNA (Figure 5D, E, E, G and 5H respectively; *P *< 0.02).

### Effect of siRNA HO-1 on canonical Wnt signaling and Wnt-responsive genes

**Figure 6:**
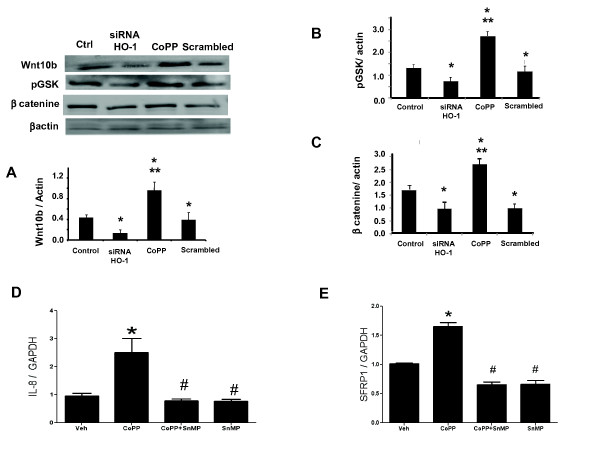
**Effect of heme oxygenase-1 (HO-1) induction and suppression on adipogenic marker expression in mesenchymal stem cell (MSC)-derived adipocytes**. (**A**-**C**) Representative immunoblots and densitometry analysis of Wnt10b, p-Glycogen synthase kinase (GSK), β-catenin levels, respectively. Data are expressed as means ± SD (*P < 0.01 vs control, **P < 0.05 vs siRNA HO-1). (**D-E**) Effect of HO-1 induction and inhibition on Wnt -responsive genes *IL-8 *and Secreted frizzled-related protein 1 (SFRP1). Data are expressed as means ± SD (*P < 0.05 vs control, ^#^*P *< 0.05 vs cobalt-protoporphyrin IX (CoPP)).

Upregulation of HO-1 by CoPP treatment increased protein expression of the canonical Wnt signaling cascade, Wnt10b, pGSK3β and β-catenin in MSC-derived adipocytes as compared to the control. To selectively assess the role of HO-1 on adiposity, HO-1 siRNA was added to cell culture with a resultant decrease in Wnt10b, pGSK3β and β-catenin levels (Figure 6A, B and 6C respectively; *P *< 0.05). Canonical Wnt signaling cascade is known to activate IL-8 and SFRP1 [46]. The levels of these Wnt responsive genes, IL-8 and SFRP1, were increased by CoPP treatment and these effects were reversed by concurrent treatment with SnMP (Figure 6D and E respectively; *P *< 0.05).

### Effect of CoPP on canonical Wnt signaling cascade during adipogenesis

**Figure 7:**
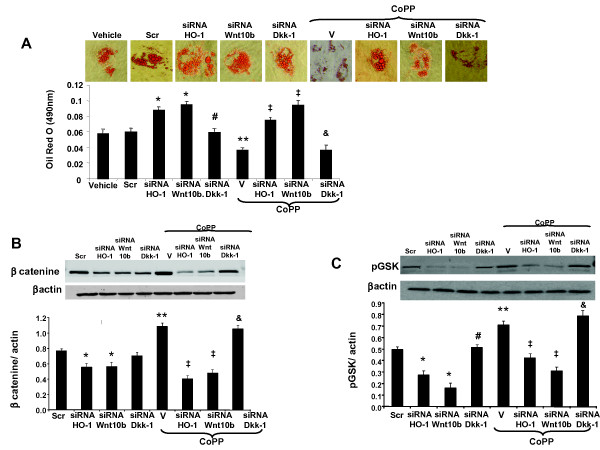
**Effect of heme oxygenase-1 (HO-1) on the Wnt canonical pathway during adipogenesis**. (**A**) Effect of inhibition of HO-1, Wnt10b and Dickkopf 1 (Dkk1) using respective siRNA on adipocyte hypertrophy. Lipogenesis was measured as the relative absorbance of Oil Red O at day 14 after inducing adipogenesis as described in Materials and methods (mean ± SD, *P < 0.05 vs vehicle, ^#^*P *< 0.05 vs siRNA Wnt10b, **P < 0.05 vs siRNA HO-1, ^‡^*P *< 0.05 vs cobalt-protoporphyrin IX (CoPP), ^&^*P *< 0.05 vs siRNA Dkk1). (**B-C**) Effect of inhibition of HO-1, Wnt10b and Dkk1 using respective siRNA on β-catenin and p- glycogen synthase kinase (GSK) levels respectively. Data are expressed as means ± SD. (*P < 0.05 vs scramble, ^#^*P *< 0.05 vs siRNA Wnt10b, **P < 0.05 vs siRNA HO-1, ^‡^*P *< 0.05 vs CoPP, ^&^*P *< 0.05 vs siRNA Dkk1).

Our results show Oil Red O staining was increased after siRNA HO-1 treatment compared to MSCs treated with CoPP (Figure 7A). To elucidate the role of Wnt10b in the regulation of adipogenesis in MSC-derived adipocytes, we measured the effect of suppression of Wnt10b on adipogenesis, using siRNA. The addition of siRNA Wnt10b increased lipid formation compared to the vehicle (*P *< 0.05), and this effect was not significantly altered by concurrent administration of CoPP, suggesting a possible interaction between HO-1 and the Wnt10b pathway. Wnt antagonist, Dickkopf 1 (Dkk1), is secreted by human pre-adipocytes and promotes adipogenesis [47]. Further our results show that knockdown of Wnt antagonist Dkk1 by siRNA showed significant reduction in lipid accumulation compared to siRNA Wnt10b, and more importantly, administration of CoPP further significantly deceased lipogenesis (*P *< 0.05; Figure 7A).

The protein expression of β-catenin and phosphorylated glycogen synthase kinase (GSK3)β was measured to study the effect of Wnt10b inhibition by using siRNA (Figure 7B and 7C respectively). Our results showed decreased expression of β-catenin and pGSK3β when MSCs were treated with Wnt10b siRNA compared to the control (*P *< 0.05) and this effect was not reversed by concurrent treatment with CoPP. We next determined whether inhibition of Dkk1 using siRNA affected these adipogenic markers. Our results further showed that β-catenin and pGSK3β levels were increased compared to the MSCs treated with siRNA Wnt10b. More importantly, concurrent administration of CoPP significantly increased the gene expression of β-catenin and pGSK3β (*P *< 0.05) compared to the cells treated with siRNA Dkk1.

## Discussion

This study demonstrates that the effects of HO-1 induction in a cell-based model of adipogenesis are dependent upon activation of the Wnt canonical signaling pathway. HO-1 induction has reduced body weight and adiposity [25,48] and improved the metabolic profile in animal models of obesity [24,49]. It has also been shown that upregulation of HO-1 reduces adipogenesis in cell cultures [34]. We show here in a cell-based model of adipogenesis (MSCs) that HO-1 induction mediates the recruitment of the Wnt canonical cascade, and entails reduced lipid accumulation comprised of smaller healthier adipocytes, reduced inflammation and improved adipokine secretion.

Differentiation of pre-adipocytes into adipocytes is regulated by a balance of transcriptional factors that can both positively and negatively influence differentiation. This is reflected by the appearance of various early, intermediate and late mRNA/protein markers and triglyceride accumulation. Several reports describe an association between adipogenesis and Wnt signaling in the regulation of adult tissue homeostasis and remodeling [50,51]. Wnt10b is an endogenous regulator of adipogenesis that maintains pre-adipocytes in an undifferentiated state and functions as an adipogenic switch. The activation of Wnt10b contributes to the inactivation/phosphorylation of GSK3β and consequently, elevated level of β-catenin; it is the molecular node of the canonical Wnt signaling pathway. When Wnt signaling is active, GSK3 is inhibited. Conversely, when Wnt signaling is suppressed, GSK3 phosphorylates β-catenin and targets it for ubiquitin-mediated degradation [11]. Our results describe a critical link between the anti-adipogenic effects of HO-1 and stimulation of the Wnt canonical pathway. Two lines of evidence characterize this relationship: First, HO-1 induction in CoPP-treated cells was accompanied by increased levels of Wnt10b and its associated signaling mediators, namely, phosphorylated GSK3β and β-catenin. The concurrent reduction in adipocyte size and lipid accumulation establishes a link between this phenotype, that is, HO-1 induction and Wnt signaling. Second, siRNA-mediated downregulation/inhibition of Wnt10b prevented HO-1 from reducing adipocyte hypertrophy in MSCs. Further our results show that inhibition of Dkk1 by siRNA decreased lipogenesis, and this effect was further enhanced by concurrent administration of CoPP. These observations indicate that activation of the Wnt canonical pathway plays a role in the prevention of adipocyte hypertrophy and the promotion of smaller healthier adipocytes in MSCs undergoing HO-1 induction. The precise molecular mechanism linking HO-1 to activation of Wnt signaling is unclear. However, the restoration of the redox environment as a result of HO-1 induction [37] could contribute to activation of Wnt signaling. In this regard, it should be noted that chronic oxidative stress has been shown to suppress the Wnt canonical pathway [52,53] while enhancing lipid accumulation and hypertrophy in MSC-derived adipocytes. Furthermore, our studies show that upregulation of HO-1and increased HO activity lead to increased levels of the Wnt-responsive genes, *IL-8 *and *SFRP1*, which was reversed by the HO-1 inhibitor, SnMP. Thus, these results substantiate our hypothesis that Wnt10b may be considered an HO-1 gene target that when increased, ultimately results in a reduction in adipocyte hypertrophy.

Activation of Wnt/β-catenin signaling maintains pre-adipocytes in an undifferentiated state through inhibition of the adipogenic transcription factors, C/EBPα and PPARγ [15-17]. C/EBPα and PPARγ have been shown to activate adipocyte-specific genes and are involved in the growth arrest that is required for adipocyte differentiation. Our results show that the increased expression of HO-1 resulted in either maintaining pre-adipocytes in the undifferentiated state or slowed down this process, presumably through activation of Wnt/β-catenin and inhibition of C/EBPα and PPARγ levels. Pref-1 has also been shown to participate in maintaining the pre-adipose phenotype. A decrease in Pref-1 expression is observed during adipocyte differentiation. In adipose tissue, Pref-1 is specifically expressed in pre-adipocytes but not in adipocytes and thus, is used as a pre-adipocyte marker [54]. In concordance with these observations, our results showed that upregulation of HO-1 increased Pref-1 expression, suggesting that HO-1 decreased adipocyte differentiation. Pref-1 prevents lipid accumulation and expression of adipocyte transcription factors such as PPARγ and C/EBPα, as well as other late adipocyte markers, including FA synthase and FABP4/aP2 [54]. *CD36 *and *aP2 *are PPARγ target genes and their mRNA levels were significantly increased during adipocyte differentiation, leading to increased lipid storage and lipogenesis [55]. Lipid accumulation of avian adipocytes is mainly dependent upon the FA transmembrane uptake process mediated by membrane proteins, such as fatty acid translocase (FAT/CD36) [45]. Our studies show that increased HO-1 gene expression decreased aP2 and CD36 levels, suggesting that HO-1 mediated increase of Wnt10b could inhibit fatty acid accumulation and lipogenesis. Our study also showed that upregulation of HO-1 is associated with increased adiponectin levels and decreased inflammatory cytokine, TNFα. Increased pro-inflammatory and reduced anti-inflammatory cytokines reflect the functional consequences of upregulation of HO-1 in MSC-derived adipocytes [34]. Smaller adipocytes are considered to be healthy, insulin-sensitive adipocytes that are capable of producing adiponectin [48]. In light of this evidence, elevation of adiponectin along with suppression of TNFα synthesis by adipocytes cultured in the presence of HO-1 induction complements the effect of the latter on adipocyte size. Together, these findings implicate the role of HO-1-Wnt signaling in bringing about reduced lipid accumulation and improved adipocyte function in MSC-derived adipocytes. Wnts, β-catenin and Shh, are essential to regulate the conversion of pre-adipocytes to adipocytes [16,47]. In this regard, we also examined Shh, which potentially works upstream of these known differentiation factors to reduce adipogenesis [8,27]. Upregulation of HO-1 increased Shh protein expression, which was reversed by siRNA of HO-1, confirming its role in decreasing adipocyte hypertrophy.

Our results show that the increase in Wnt10b in parallel with the increase in *HO-1 *gene expression by CoPP was associated with a significant reduction in levels of Peg-1/Mest. A decrease in Peg-1/Mest is beneficial in the control of obesity, since upregulation of Peg-1/Mest occurs in obese adipose tissue in several models of obesity [21,56]. Our data demonstrate that the induction of HO-1 was effective in suppressing adipocyte differentiation, as evidenced by an increase in the canonical Wnt cascade and a decrease in Peg-1/Mest. These effects were reversed by blocking *HO-1 *gene expression by siRNA, further demonstrating that HO-1 mediated-increase in Wnt10b, and decrease in Peg-1/Mest resulted in the maintenance of pre-adipocytes in their undifferentiated state with the slowing of the differentiation process. Taken together, these observations provide compelling evidence that HO-1-mediated increase in Wnt signaling and its associated genes modulate adipogenesis.

## Conclusions

**Figure 8:**
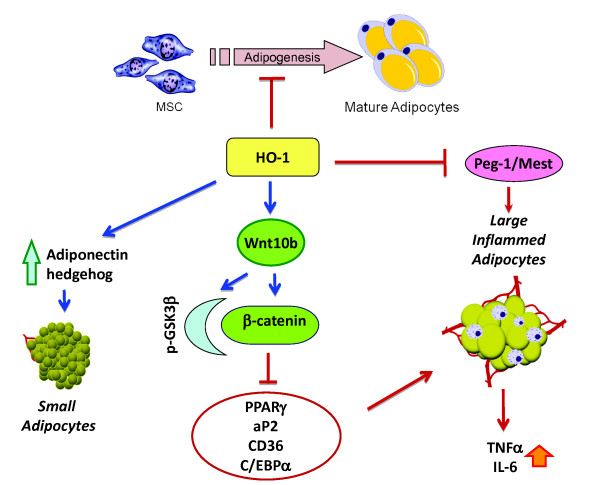
**Proposed mechanism demonstrating the interplay of heme oxygenase 1 (HO-1) and the Wnt canonical pathway in the modulation of adipogenesis in mesenchymal stem cells (MSC)-derived adipocytes**. HO-1 suppresses adipocyte differentiation by increasing expression of key regulators including Wnt10b, p-glycogen synthase kinase (GSK), β-catenin and Sonic hedgehog (shh) and decreasing expression of adipogenic transcription factors CCAAT/enhancer binding protein a (CEBP)α, peroxisome proliferator-activated receptor (PPAR)γ and fatty-acid-binding protein (aP2). These effects led to decrease in lipid accumulation and increase of pre-adipocytes and healthy adipocytes that produce cytoprotective adipokines, such as adiponectin.

In conclusion, as represented in the schematic (Figure 8), our novel study demonstrates that upregulation of *HO-1 *gene expression and increased HO activity decreased adipocyte differentiation, with an associated increase in the number of small healthy lipid droplets via interplay of the Wnt signaling cascade. This is evident by the fact that increased HO-1 expression and HO activity decreased adipocyte hypertrophy, decreased TNFα levels, increased adiponectin level and increased expression of the genes central to the canonical Wnt signaling cascade. Thus, crosstalk between HO-1 and Wnt10b could be employed therapeutically to suppress adipogenesis, and therefore constitutes an attractive drug development target to combat obesity-associated metabolic complications.

## Abbreviations

aP2: fatty-acid-binding protein 4 (FABP4); BODIPY: boron-dipyrromethene; C/EBP: adipogenic transcription factors CCAAT/enhancer binding protein a; CoPP: cobalt-protoporphyrin IX; DAPI: 4',6-diamidino-2-phenylindole; Dkk-1: Dickkopf 1; EDTA: ethylenediaminetetraacetic acid; EET: epoxyeicosatrienoic acids; FA: fatty acid; FAT/CD36: fatty acid translocase; FACS: fluorescence-activated cell sorter; FBS: fetal bovine serum; GC/MS: gas chromatography/mass spectrometry; GSK3 β: glycogen synthase kinase 3β; HMW: high molecular weight; HO-1: heme oxygenase-1; IL: interleukin; LDH: lactic dehydrogenate; Mest: Mesoderm-specific transcript; MSC: mesenchymal stem cells; NADPH: nicotinamide adenine dinucleotide phosphate-oxidase; OD: optical density; PBS: phosphate-buffered saline; PCR: polymerase chain reaction; Peg 1: Paternally expressed 1; PMSF: phenylmethylsulfonyl fluoride; PPAR: peroxisome proliferator activator receptor; Pref-1; Pre-adipocyte factor-1; SE: standard error; SFRP1: Secreted frizzled-related protein 1; Shh: Sonic hedgehog; SnMP: tin (stannic)-mesophorphyrin IX; TNF: tumor necrosis factor; Wnts: Wingless-type.

## Competing interests

The authors declare that they have no competing interests.

## Authors' contributions

LV contributed equally to this work performing cell culture and HO levels and contributed in conception and design. KS contributed equally to this work, drafted the manuscript and repeated all work done by LV to confirm the results. D-HK performed Oil red stain and measurements. NP designed the siRNA, treatments, and harvested stem cells from culture. MM performed western blots for signaling. LB performed the cytotoxic assay. SJP was involved in design and input for the significance for clinical application. NGA, PI, designed, accumulated data, compared figures and examined various experiments. JIS revised the discussion and input to redraw several figures and scheme. All authors read and approved the final manuscript for publication.
